# Anti-Tubercular Activity of Substituted 7-Methyl and 7-Formylindolizines and In Silico Study for Prospective Molecular Target Identification

**DOI:** 10.3390/antibiotics8040247

**Published:** 2019-12-03

**Authors:** Katharigatta N. Venugopala, Christophe Tratrat, Melendhran Pillay, Fawzi M. Mahomoodally, Subhrajyoti Bhandary, Deepak Chopra, Mohamed A. Morsy, Michelyne Haroun, Bandar E. Aldhubiab, Mahesh Attimarad, Anroop B. Nair, Nagaraja Sreeharsha, Rashmi Venugopala, Sandeep Chandrashekharappa, Osama I. Alwassil, Bharti Odhav

**Affiliations:** 1 Department of Pharmaceutical Sciences, College of Clinical Pharmacy, King Faisal University, Al-Ahsa 31982, Saudi Arabia; ctratrat@kfu.edu.sa (C.T.); momorsy@kfu.edu.sa (M.A.M.); mharoun@kfu.edu.sa (M.H.); baldhubiab@kfu.edu.sa (B.E.A.); mattimarad@kfu.edu.sa (M.A.); anair@kfu.edu.sa (A.B.N.); sharsha@kfu.edu.sa (N.S.); 2 Department of Biotechnology and Food Technology, Durban University of Technology, Durban 4001, South Africa; odhavb@dut.ac.za; 3 Department of Microbiology, National Health Laboratory Services, KZN Academic Complex, Inkosi Albert Luthuli Central Hospital, Durban 4001, South Africa; melendhra.pillay@nhls.ac.za; 4 Department of Health Sciences, Faculty of Science, University of Mauritius, Réduit 80835 80832, Mauritius; f.mahomoodally@uom.ac.mu; 5 Department of Chemistry, Indian Institute of Science Education and Research Bhopal, Indore By-pass Road, Bhauri, Bhopal 462 066, Madhya Pradesh, India; subhb@iiserb.ac.in (S.B.); dchopra@iiserb.ac.in (D.C.); 6 Department of Pharmacology, Faculty of Medicine, Minia University, El-Minia 61511, Egypt; 7 Department of Public Health Medicine, University of KwaZulu-Natal, Howard College Campus, Durban 4001, South Africa; venugopalar@ukzn.ac.za; 8 Institute for Stem Cell Biology and Regenerative Medicine, NCBS, TIFR, GKVK, Bellary Road, Bangalore 560 065, India; 9 Department of Pharmaceutical Sciences, College of Pharmacy, King Saud bin Abdulaziz University for Health Sciences, Riyadh 11481 11543, Saudi Arabia; wassilo@ksau-hs.edu.sa

**Keywords:** indolizines, *Mycobacterium tuberculosis*, multi-drug resistance, molecular modeling, multi-component reaction, whole-cell anti-TB screening

## Abstract

Novel series of diversely substituted indolizines were designed, synthesized, and evaluated for their in vitro anti-mycobacterial activity against H37Rv and multi-drug-resistant (MDR) strains of *Mycobacterium tuberculosis* (MTB). Many compounds exhibited significant inhibitory activity against MTB H37Rv strains. Indolizines 2d, 2e, and 4 were also found to be active against MTB clinical isolates with multi-resistance to rifampicin and isoniazid. Indolizine **4** was identified as the most promising anti-mycobacterial agent, displaying minimum inhibitory concentration (MIC) values of 4 and 32 μg/mL against H37Rv and MDR strains, respectively. Furthermore, an in silico study was carried out for prospective molecular target identification and revealed favorable interactions with the target enzymes CYP 121, malate synthase, and DNA GyrB ATPase. None of the potent molecules presented toxicity against peripheral blood mononuclear (PBM) cell lines, demonstrating their potentiality to be used for drug-sensitive and drug-resistant tuberculosis therapy.

## 1. Introduction

*Mycobacterium tuberculosis* (MTB) is responsible for the development of the disease tuberculosis (TB), which is airborne and highly contagious. TB is one of the leading causes of death worldwide and is produced by a single infectious agent. According to the 2019 global tuberculosis report, TB resulted in nearly 1.5 million deaths, including 251,000 people that were HIV-positive [1]. The increase in the prevalence of multi-drug-resistant (MDR)-TB [2] and extensively drug-resistant (XDR)-TB [3] has increased the requirement for more effective therapeutic regimens with fewer side effects. Treating the MDR-TB and XDR-TB has proven to be more challenging, as second-line drugs have largely become less effective [4]. This problem has worsened given the emergence of totally drug-resistant (TDR) strains of MTB [5]. TDR cannot be treated using the currently available anti-TB drugs. According to the literature search from the last 40 years of academic and pharmaceutical drug discovery industry inventions, the US Food and Drug Administration (US FDA) in December 2012 approved only bedaquiline as the first novel anti-TB drug for the treatment of MDR-TB [6], while the European Medicine Agency in late 2013 approved delamanid as the second anti-TB agent [7].

**Figure 1:**
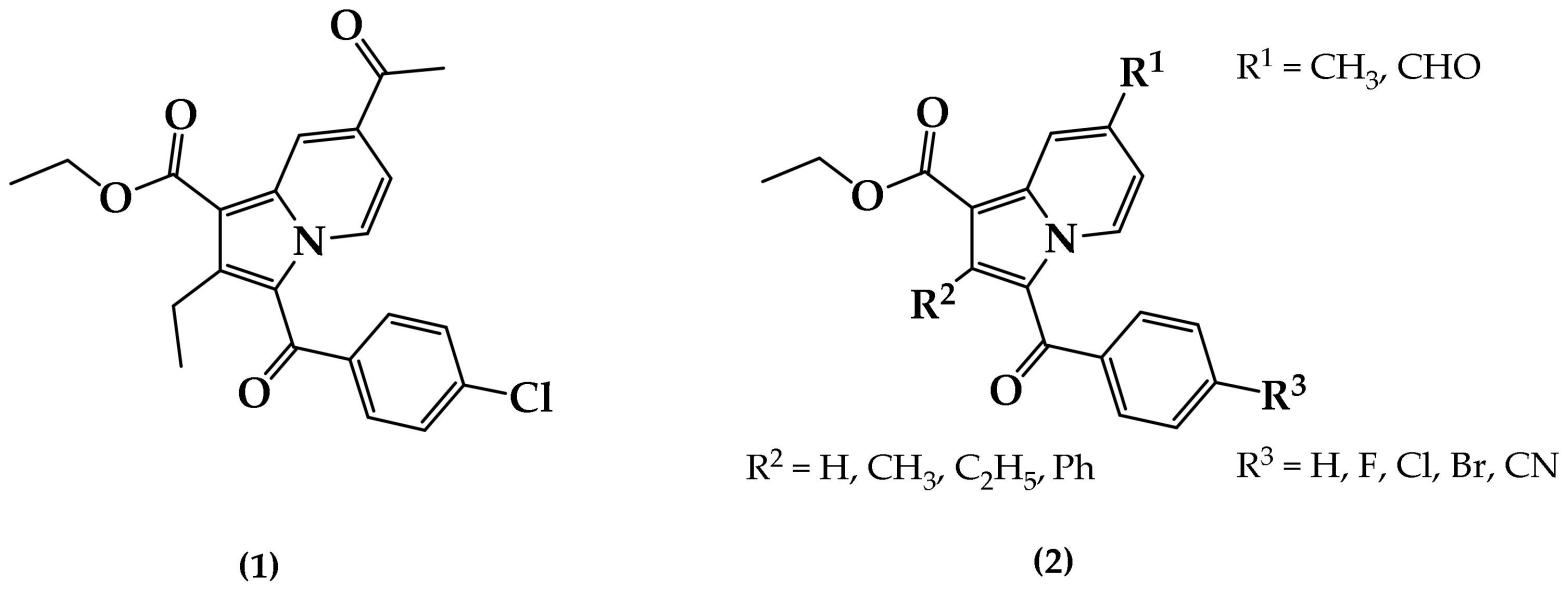
Chemical structure of anti-tuberculosis (TB) indolizine compound (**1**) and the proposed poly-functionalized indolizines (**2**) as potential anti-TB agents.

Indolizine represents an interesting heterocyclic scaffold in which nitrogen belongs to both fused six- and five-membered rings. It is a well-known pharmacophore responsible for various promising pharmacological properties. For instance, indolizines were found to exhibit analgesic [8], anticancer [9,10], antidiabetic [11], antihistaminic [12], anti-inflammatory [13,14], antileishmanial [15], antimicrobial [16], antimutagenic [17], antioxidant [18], antiviral [19], larvicidal [20,21], and herbicidal [22] activities. However, the anti-TB activity of indolizine is poorly documented in the literature [23,24,25,26]. Recently, our group started investigating multifunctionalized indolizine pharmacophores for their chemistry, structural elucidation, and pharmacological properties, including their anticancer properties [10], cyclooxygenase-2 (COX-2) inhibition properties [14,27], and larvicidal activity against *Anopheles arabiensis* [21]. In continuation of our effort to identify novel potent anti-TB agents of cyclic depsipeptides [28] and heterocyclic origin [29,30,31,32,33,34], we previously identified a series of 7-acetyl indolizines exhibiting interesting anti-mycobacterial activity. A preliminary structure–activity relationship was determined to demonstrate that the indolizine (1) displayed the most potent activity at 11 μg/mL against both H37Rv and MDR strains of MTB (Figure 1) [31]. In the present investigation, we explore the impacts of the functionalization at positions 2 and 7 of a novel series of 3-substituted benzoylindolizine (2) on the anti-tubercular activity against H37Rv and MDR strains of MTB. The whole-cell anti-TB screening process will help to identify the key substituents responsible for the biological activity, thereby facilitating the discovery of potential molecular target(s) through a computational docking study.

## 2. Results and Discussion

### 2.1. Chemistry

**Scheme 1:**
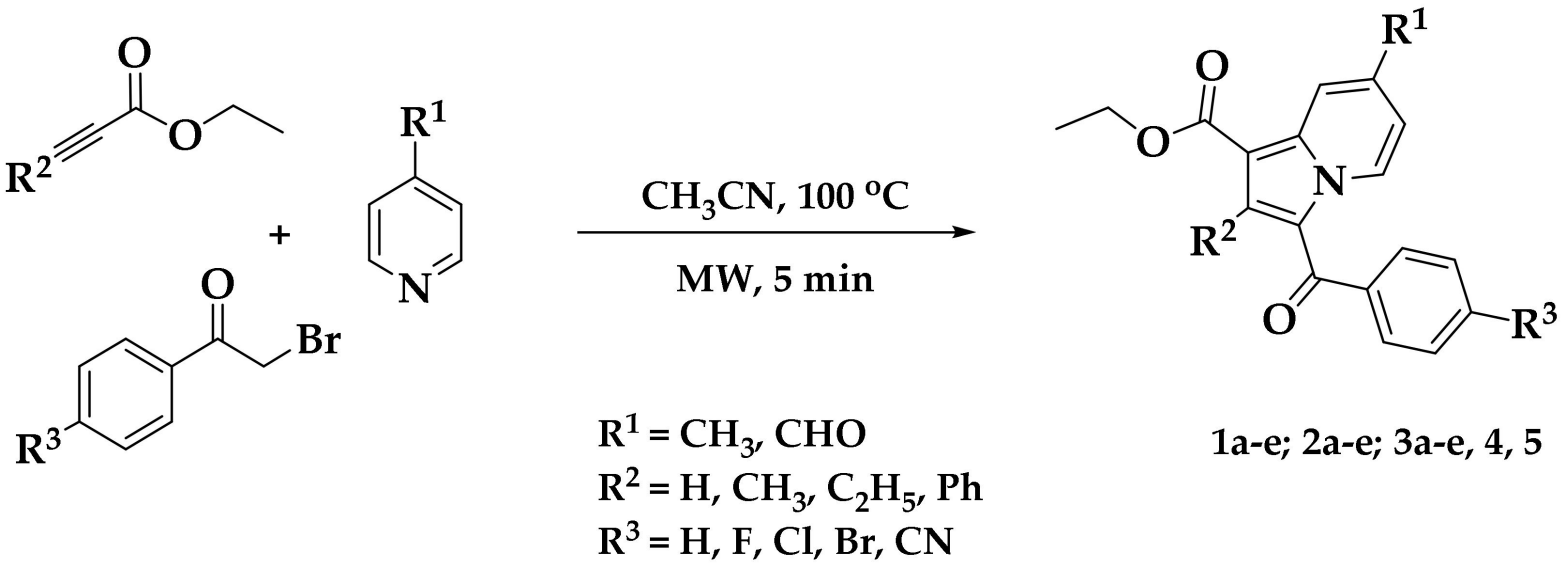
Microwave-assisted multicomponent reaction (MCR) for the synthesis of poly-functionalized indolizines.

Multicomponent reactions (MCRs) have gained considerable prominence in the drug-discovery process in both academia and industry. MCRs provide medicinal chemists with the opportunity to rapidly access novel scaffolds with a high degree of structural functionality and complexity, accelerating lead compound identification [21]. MCRs are associated with various advantages such as single operation, synthetic efficiency, and a diversity of inputs, both economic and ecological. We recently reported an efficient, convenient, one-pot MCR for the preparation of diversely substituted indolizines at high yields [14]. The reaction involved condensing three components: substituted pyridine, substituted bromoacetophenone, and ethyl propiolate/ethyl but-2-ynoate/ethyl hex-2-ynoate/methyl 3-phenylpropiolate. This method was highly convergent and flexible, allowing functionalization construction. The use of this synthetic development process allowed us to prepare a variety of indolizine derivatives. The chemical synthesis of the indolizine **1a–e**, **2a–e**, and **3a–e** were achieved by employing a one-pot MCR using a microwave method (Scheme 1) [21]. This method was ecofriendly, and the yield of the test samples was found to be in the range of 85–94%.

Two novel compounds, 7-formyl-2-methylindolizine derivative 4 and 7-methyl-2-phenylindolizine derivative 5, were synthesized using the reported microwave method, and their chemical structures were confirmed by FT–IR, nuclear magnetic resonance (NMR; ^1^H and ^13^C), LC–MS, and elemental analysis (Table 1).

### 2.2. Anti-Tubercular Activity

All synthesized indolizine derivatives (1a–e, 2a–e, 3a–e, 4, and 5) were assessed for in vitro whole-cell anti-TB properties against H37Rv and MDR strains of *Mycobacterium tuberculosis*. The results are expressed as MIC (µg/mL) and are summarized in Table 2. Out of 17 compounds screened for their anti-TB properties, only three compounds, 2d, 2e, and 4, exhibited anti-TB property against both H37Rv and MDR strains of *Mycobacterium tuberculosis*. However, the test compounds 2c, 3a, and **4** exhibited activities against only H37Rv strains of *Mycobacterium tuberculosis*. It was also observed from the in vitro results of test compounds 1a–e, 2a–e, and 3a–e that two methyl groups at the second and seventh positions of the indolizine nucleus, as well as electron-withdrawing functional groups at the *para* position of the benzoyl ring, which is at the third position of indolizine nucleus, were important for exhibiting anti-TB activity against H37Rv and MDR strains of *Mycobacterium tuberculosis*. However, compound 5, in which the phenyl ring was in place of a methyl group at the second position of the indolizine nucleus, only displayed modest activity against susceptible H37Rv strains of *Mycobacterium tuberculosis*. It was interesting to note that electron withdrawing halogen atoms—such as chlorine, bromine, and fluorine—at the *para* position of the benzoyl ring, which is at the third position of the indolizine nucleus and ethyl group at the second position of indolizine nucleus, did not favor anti-TB activity against MDR strains of *Mycobacterium tuberculosis*. Lastly, compound 4, containing a formyl functional group at the seventh position of the indolizine nucleus and a bromo group at the *para* position of the benzoyl moiety located at third position of indolizine ring, was found to exhibit the most promising anti-mycobacterial activity by displaying MIC at 4 µg/mL and 32 µg/mL against H37Rv and MDR strains of *Mycobacterium tuberculosis*, respectively. This finding strongly corroborates the reported preliminary structure–activity result of the 7-acetyl indolizine series [31], in which the presence of a polar group (carbonyl) at the seventh position of the indolizine ring was an important criteria for retaining anti-mycobacterial inhibition.

### 2.3. Safety Studies

To assess the toxicity of the most promising anti-TB compounds 2d, 2e, and 4 from the series, an MTT assay was conducted. It was found that none of the test compounds exhibited cytotoxicity up to 500 µg/mL across peripheral blood mononuclear (PBM) cell lines.

### 2.4. Molecular Modeling

To highlight the bioactivity of the indolizines against susceptible strains of *M. tuberculosis* (H37Rv), we explored the binding capability of newly synthesized indolizines with different molecular targets in an attempt to identify one or several plausible mechanisms of action. Many MTB targets were investigated in computational molecular modeling studies: Pantothenate (PDB 3IVX), Pank (PDB 4BFU), InhA (PDB 1P44), DprE1 (PDB 4P8Y), BioA (PDB 4XJO), PtpB (PDB 1YWF), β-Lactamase (PDB 4X6T), GyrB ATPase (PDB 4B6C), Malate synthase (PDB 5CBB), and Cytochrome P450 CYP 121 (PDB 5OP9). Our efforts were concentrated on establishing a correlation between the bioactivity and binding affinity of the promising test compounds. The presence of a substituent at position 7 of indolizine seems to be prominent for *M. tuberculosis* inhibition. Indeed, functionalization at position 7 of the indolizine by a polar group (formyl) displayed better activity when compared to the nonpolar group (methyl). This finding indicated that the carbonyl functional group played an important role in the molecular interaction with the receptor. Our computational input revealed that, among all investigated target enzymes, only three receptors—CYP121, Malate synthase, and GyrB ATPase—were found to be consistent in terms of their binding affinity and their biological activities with the indolizine derivatives. The docking results are reported in Table 3, which displays the docking energy and the interactive residue of the selected molecular targets. The preferred binding mode for each target is discussed to support the observed in vitro activity against H37Rv of MTB.

### 2.5. Molecular Docking Analysis with CYP121 Receptor

The binding study of indolizines into the CYP121A1 (PDB 5OP9) receptor showed higher docking energy for compounds 2c–e and 4 (Table 3). Most indolizines displayed similar binding modes to that of compound 4, except for compounds 3b and 3e. The predicted binding mode indicated that the methyl at position 2 of the indolizine was pointed toward the pocket made by residue PHE 168. A strong H-bond involving the carbonyl of benzoyl and the NH of the GLN 385 residue, with an average distance of 2 Å, was observed. As for compound 4, the presence of the formyl group at position 7 of the indolizine contributed to establishing a moderate H-bond interaction with the NH of the amino acid residue ASN 85 (average bond length interaction: 2.4 Å). Two types of π bond interactions were observed from the binding pose: either a π–π stacking interaction between the indolizine ring and the phenyl of the residue PHE168, or a sulfur–π interaction with the indolizine ring and the methionine residue MET 62.

**Figure 2:**
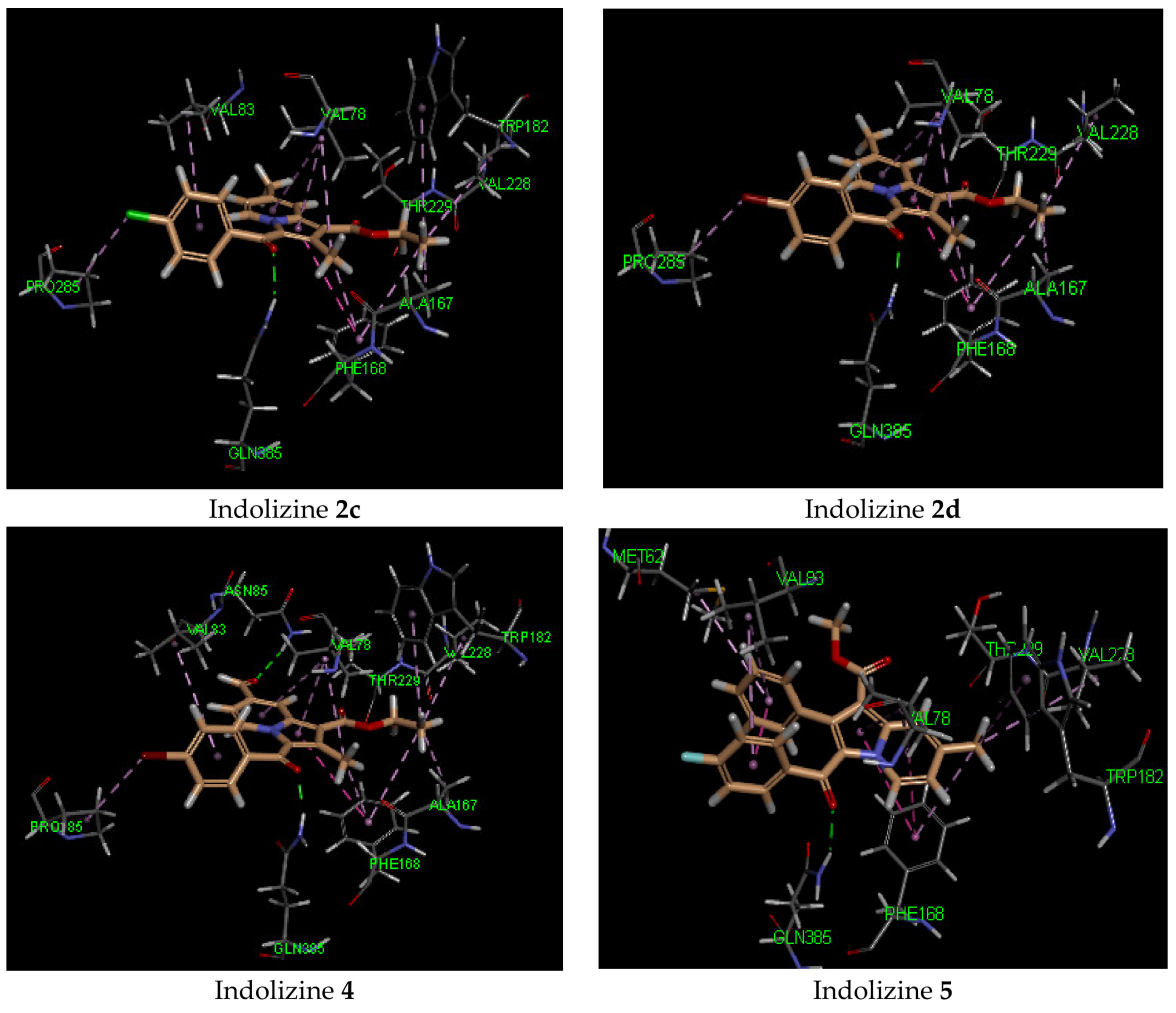

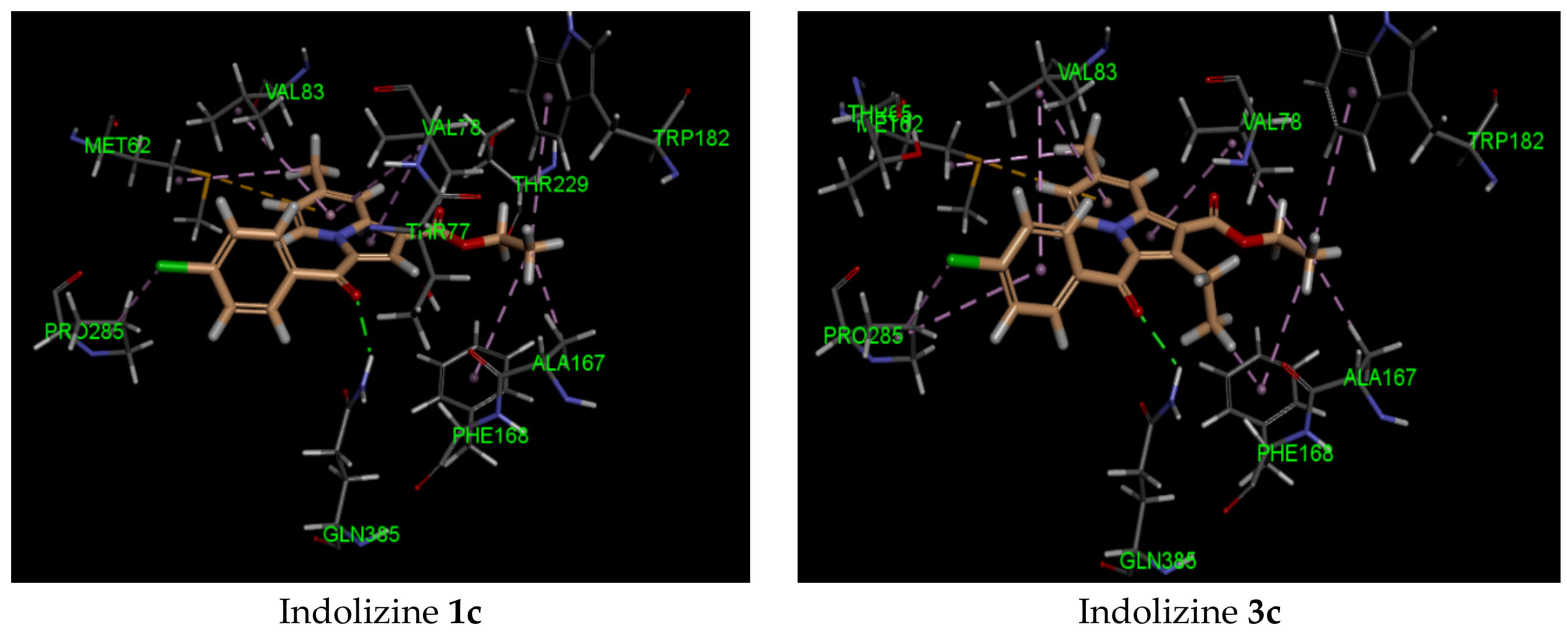
Predicted interaction patterns of compounds 1c, 2c, 2d, 3c, 4, and 5 (salmon-filled spheres) into the CYP121 domain (PDB 5OP9). The molecular interactions are represented with a green dotted line for the H-bond, a magenta dotted line for the π–π stacking interaction, and a violet dotted line for the hydrophobic contact.

It is noteworthy that the hydrophobic pi interaction PHE 168 seemed to be critical for retaining inhibitory activity. Indeed, the indolizine derivatives 2c–e and 4 were involved in such interactions, but the inactive compounds such as 1c and 3c (Figure 2) were only involved in pi–alkyl interactions with the residue PHE 168 and the ethyl groups from the ester and from position 2 of indolizine. Moreover, the alkyl substituent in position 2 of the scaffold provided an alkyl–pi interaction with PHE 168 and an alkyl–alkyl interaction with the residue VAL 78, allowing improved binding affinity. Additional alkyl–pi interactions were also observed with VAL 78 and both indolizino rings, and VAL 83 with benzoyl moiety of derivatives. The halogenated derivatives allowed the formation of the hydrophobic interaction with the proline residue PRO 285 for compounds 2c,d, and 4, as depicted in Figure 2. It is worth noting that the methyl group at position 7 did not seem to contribute to the hydrophobic interaction with the CYP 121 domain.

In contrast, indolizine (4), bearing a polar group at position 7, represented the most active compound in the series, demonstrating higher docking scores. It was predicted to bind in a similar fashion to that of 2c within the binding pocket via an additional H-bond interaction with the formyl group of the ligand with NH of the residue ASN 185. As for compound 5, which was substituted in position 2 by a phenyl group, it showed a similar docking score as its congeners, but displayed a different orientation in the binding domain. The indolizine 5 appeared to bind flipped over the benzoyl ring, in which the benzo ring of indolizine was pointed toward the residue PHE 168.

The predicted binding pose indicated the establishment of a hydrogen bond between the benzoyl carbonyl group and the residual GLN 385. The indolizine moieties were involved in π–π stacking interactions with amino acid PHE 168. The conformation of 5 within the active site adopted a similar conformation to that of the crystallographic structure. In both cases, the benzoyl ring and the phenyl ring at position 2 interacted with each other through a π–π stacking interaction. The intermolecular force distance was predicted to be 3.64 Å from the docking pose, which was consistent with that of the crystallography data (from 3.46 to 3.54 Å). Additionally, the methyl group at position 7 formed strong hydrophobic contacts with residues PHE 168, TRP 182, and VAL 228. In general, the docking results suggested that the bioactive indolizines were involved in hydrophilic contacts with residues GLN 385 and ASN 185, and in hydrophobic contacts with VAL 78, VAL 82, VAL 83, ALA 167, and PHE 168. The observed interaction pattern was in accordance with the interactions reported for known CYP121 inhibitors [35,36].

### 2.6. Molecular Docking Analysis with Malate Synthase Receptor

**Figure 3:**
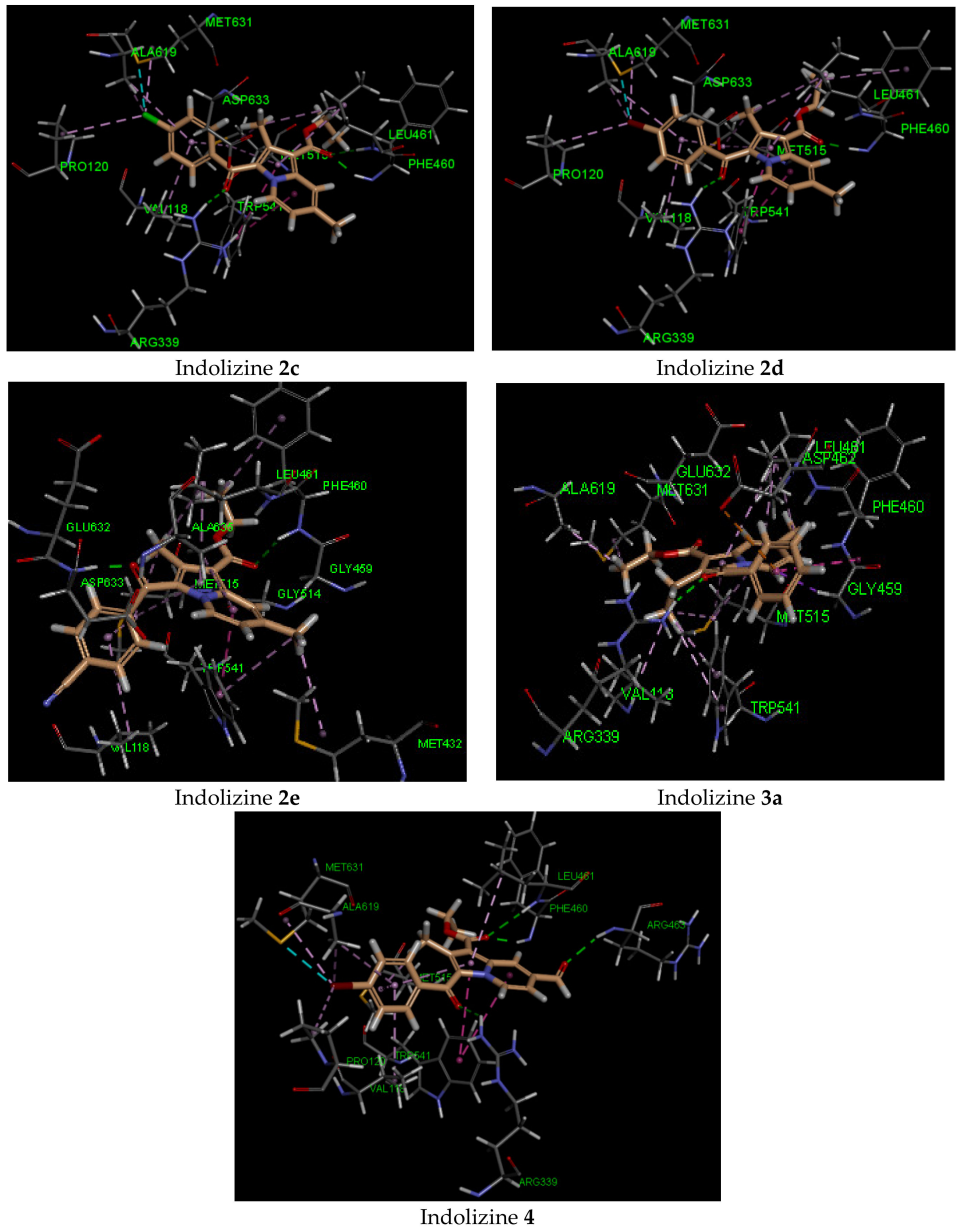
Predicted interaction pattern of 2c, 2d, 2e, 3a, and 4 (salmon-filled spheres) in the malate synthase domain (PDB 5CBB). The molecular interactions are represented with a green dotted line for the H-bond, with a magenta dotted line for the π–π staking interaction, with an orange dotted line for the ion–π staking interaction, and with a violet dotted line for the hydrophobic contact.

The docking findings revealed that compounds 2c–e and 4 displayed a stronger binding affinity to the receptor by showing stronger docking energy scores (Table 3). Among them, the most active compound (4) showed the strongest negative binding energy, which was consistent with the observed inhibitory activity. The predicted docking poses indicated that the hydrophilic contacts with the residues ARG 339, PHE 460, LEU 461, ASP 462, and ARG 463, as well as the hydrophobic contacts with TYR 541, PRO 120, VAL 118, MET 515, and MET 631 were involved. The predicted binding mode of the active compounds showed that 2c,d and 4 adopted identical conformation within the active domain (Figure 3). The benzoyl ring and the azino ring were oriented by approaching the amino acid ARG 339. The latter was involved in moderate H-bonding interactions (2.7 Å) with the guanidine moiety and the benzoyl carbonyl. It was also predicted that a strong H-bond would form with the NH residue of PHE 460 and with the ester carbonyl, with an average distance of 2 Å. Another moderate H-bond interaction with an ester carbonyl and LEU 461 was only found for 2c and 4.

We also observed that the residue ARG 463 through the NH amino acid moiety participated moderately in the H-bond interaction with the formyl group of indolizine 4 and might be responsible for the observed higher binding affinity. As for hydrophobic interactions, the indolizine ring interacted with the residue TRYP 541 via π-staking interaction, and the halogen substituent (2c,d and 4) engaged in binding with residues ALA 619, PRO 120, and MET 631. The alkyl–alkyl interaction was only observed for 2-alkyl substituted indolizines with the residue MET 515. Unfortunately, the methyl group in position 7 for compounds 2c,d, and 4 was not involved in any hydrophobic interactions. However, derivatives 2e and 3a demonstrated MTB inhibition activity and were found to form hydrophobic interactions with methyl at position 7, as well as with residues MET 432, TRYP 541, and LEU 461, respectively. Indolizine 2e developed two H-bonds with residues ASP 633 and PHE 460 that occurred with benzoyl carbonyl and ester carbonyl, respectively, while **3a** formed only one H-bond with residue ARG 339 and benzoyl carbonyl. An interesting binding feature observed for 3a was the formation of an anion–π stacking interaction between the benzoyl group and the carboxylate ion of the ASP 462 amino acid. Finally, indolizine **5**, bearing a phenyl group at position 2, gave a weak energy docking score (−18 kcal/mol), indicating that the ligand–receptor interaction was not favorable, although the unusual docking pose revealed the formation of three H-bond interactions with ARG 339, ARG 463, and ASP 633.

### 2.7. Molecular Docking Analysis with GyrB ATPase Receptor

The docking study was carried out using a *M. smegmatis* DNA GyrB ATPase receptor (PDB 4B6C), an organism different from that used for biological evaluation (H37Rv). A putative model of the *M. tuberculosis* H37Rv GyrB ATPase binding pocket was suggested using the *Escherichia coli* GyrB crystal structure [37]. The proposed interaction pattern in this model was confirmed in a computational docking study using a crystallographic structure from the *M. smegmatis* GyrB domain. Both receptors differed in terms of either the location number or the type of key amino acid residue interactions. For instance, residues ARG 121, ARG 180, and SER 208 in *M. tuberculosis* H37Rv GyrB corresponded to ARG 82, ARG 141, and THR 169, respectively, in *M. smegmatis* GyrB. Therefore, the choice to use the receptor from *M. smegmatis* for the docking study seemed to be sound when determining potential *M. tuberculosis* H37Rv GyrB inhibitors.

**Figure 4:**
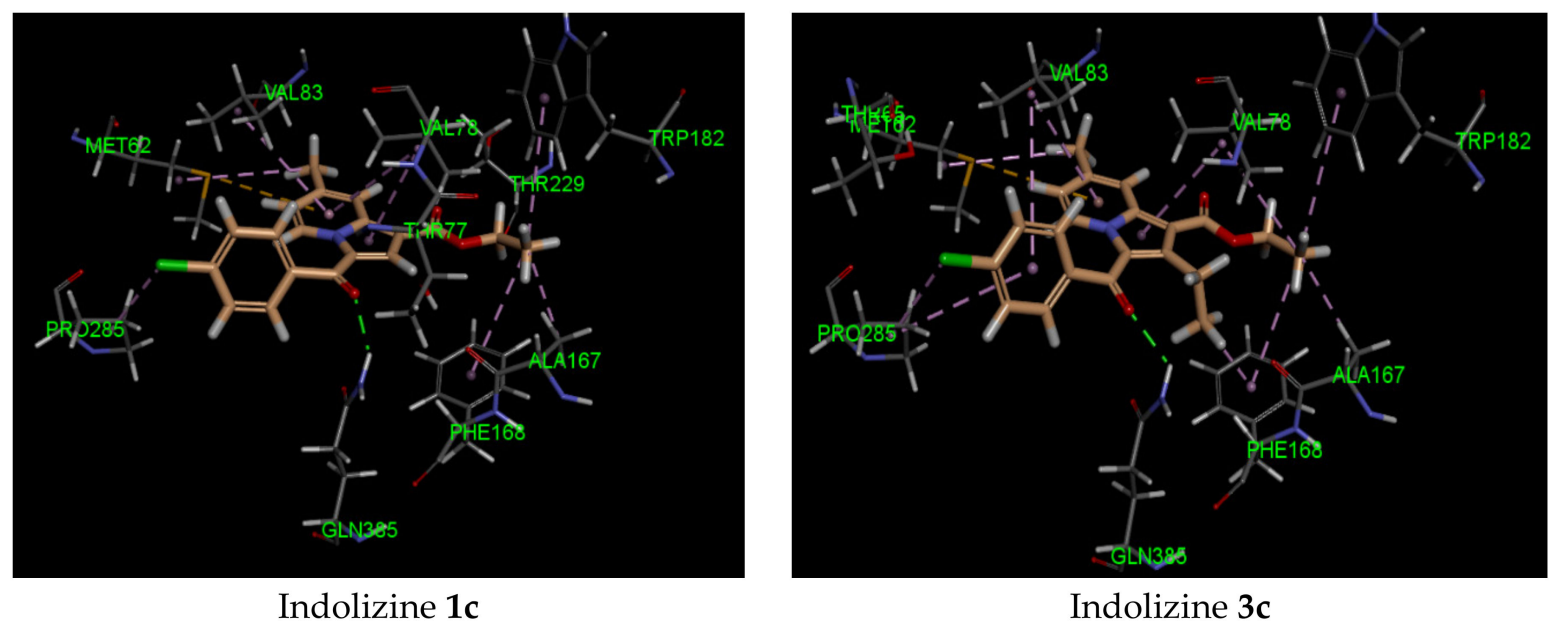
Predicted interaction patterns of 3a and 4 (salmon-filled spheres) into the DNA GyrB ATPase domain (PDB 4B6C). The molecular interactions are represented with a green dotted line for the H-bond, an orange dotted line for the ion–π interaction, and a violet dotted line for hydrophobic contact.

Based on our docking results, indolizine 4 displayed the highest docking energy, which was in agreement with the in vitro inhibitory activity (Table 3). Indeed, compound 4 displayed three H-bond interactions with the receptor, while the other derivatives were only involved with one H-bond interaction. In most cases, the GLN 102 amino acid developed a moderate H-bond with benzoyl carbonyl (average length: 2.2 Å), with the exception of compound 2e, which was involved with the ester carbonyl group. Three plausible binding modes were predicted, as presented in Figure 4. Some of these adopted the same orientation as indolizine 4. From the predicted docking pose, the indolizine structure was found above either the GLU 56 or ARG 82 residue via an anion–π or cation–π interaction, respectively, with the azino ring of indolizine. An interesting trend was observed for halogenated derivatives 2c,d, and 4 (Cl and Br): the presence of halogen induced the position of indolizine within the binding pocket by orienting the benzoyl ring toward the pocket made by the amino acid residue VAL 123. The latter formed a hydrophobic interaction with chlorine and bromine. Hence, the binding orientation of 2c,d displayed similar orientations to that of 4.

As depicted in Figure 4, two possible orientations of the indolizine derivatives were observed in DNA GyrB ATPase binding domain: either the benzo ring of indolizine was pointed toward the residue ILE 84 (compound 4) or the ester group was directed to residue ILE 84 (compound 3a). As for compound 4, the binding interaction showed involvement in the H-bond of the GLN 102, ARG 141, and GLY 83 residues with benzoyl carbonyl, ester carbonyl, and formyl carbonyl, respectively. The strongest H-bond was observed for the NH of GLY 83 and the formyl interaction (2.13 Å), while the weakest H-bond was observed for the NH of ARG 141 and ester carbonyl interaction (3.04 Å). The predicted binding mode for **3a** demonstrated a strong H-bond interaction with the amino acid GLN 102 and displayed a cation–π interaction with ARG 82 (Figure 4). This binding mode was also observed for inactive compounds 3b, 3c, and 3e. The methyl substituent in position 7 of the indolizine ring participated in a hydrophobic interaction with either ILE 84 or PRO 85. Lastly, the presence of the phenyl group in position 2 for derivative **5** had a detrimental effect on binding affinity, showing a low energy docking score.

The findings of our present molecular modeling facilitated the identification of three plausible molecular targets among investigated MTB targets. The indolizines may be regarded as potential mycobacterial CYP121 inhibitors, malate synthase inhibitors, or GyrB ATPase inhibitors with the exception of indolizine 5, which showed only favorable binding affinity for the CYP121 target enzyme. The presence of the polar group in position 7 indicated the enhancement of the binding affinity of the compound within the active site of the three receptors, as revealed in the inhibitory activity. However, additional study is needed to ascertain if one of these proteins might actually be the target of the compounds.

## 3. Materials and Methods

### 3.1. General

All chemicals were obtained from Sigma-Aldrich Co. and used without further purification. Thin-layer chromatography (TLC) was performed on silica gel (Sigma-Aldrich Co., St. Louis, MO, USA) on TLC aluminum foils using ethyl acetate and *n*-hexane (3:7) as a solvent. The samples were visualized in an iodine chamber and ultraviolet (UV) chamber. A Büchi melting-point B-545 apparatus was used to determine the melting points of the products. ^1^H and ^13^C-NMR spectra were recorded on Bruker AVANCE III 400 MHz instruments using CDCl_3_ as a solvent. Chemical shifts (*δ*) were recorded in parts per million (ppm) downfield from tetramethylsilane; then, the coupling constants (*J*) were recorded in Hertz. Splitting patterns for singlet (s), doublet (d), quartet (q), and multiplet (m) were abbreviated. The Fourier transform–infrared (FT–IR) spectra were recorded on a Shimadzu FT–IR spectrophotometer. Elemental analysis was performed using a Thermo Finnigan FLASH EA 1112 CHN analyzer. Mass spectra were recorded using the LC–MS-Agilent 1100 series with MSD (an ion trap) using 0.1% aqueous trifluoroacetic acid in an acetonitrile system on the C18-BDS column. A single-crystal X-ray diffraction study was performed using a Bruker KAPPA APEX II DUO diffractometer equipped with a CCD detector; monochromated Mo K*α* radiation (λ = 0.71073 Å) was used. Data collection was carried out at 173(2) K using an Oxford Cryostream cooling system featuring Bruker Apex II software. Except for compounds 4 and 5 from Scheme 1, the synthesis of all analogues was reported in our previous communications [14,21].

### 3.2. Synthetic Procedure for the Construction of Ethyl 3-(4-Bromobenzoyl)-7-formyl-2-methylindolizine-1-carboxylate *(4)*

A mixture of isonicotinaldehyde (107 mg, 1 mmol), 2-bromo-1-(4-bromophenyl)ethan-1-one (275 mg, 1 mmol), triethylamine (0.101 g, 1 mmol), and ethyl but-2-ynoate (112 mg, 1 mmol), in 4.5 mL of acetonitrile, was added to a 10 mL microwave tube under a nitrogen atmosphere. For about 5 min, a microwave initiator was used to irradiate the reaction mixture at 100 °C. The chemical reaction was monitored via thin-layer chromatography. The solvent of the reaction medium was removed under reduced pressure, the crude residue was diluted with water, and the aqueous layer was twice extracted with ethyl acetate, and the combined organic solvent was washed with brine solution. The organic layer was removed under reduced pressure, and the remaining residue was subjected to column chromatography using 60–120 mesh silica gel with an ethylacetate and *n*-hexane solvent system to afford 0.3551 g (86% yield) of ethyl 3-(4-bromobenzoyl)-7-formyl-2-methylindolizine-1-carboxylate 4 (Table 1). Appearance: Yellow crystalline compound; FT–IR (KBr, cm^–1^): 3058.89, 1652.88, 1608.52, 1587.31, 1541.02, 1487.01, 792.69; ^1^H-NMR (400 MHz CDCl_3_) *δ* = 9.40–9.39 (m, 1H), 9.04 (s, 1H), 7.91–7.90 (m, 2H), 7.63–7.61 (m, 2H), 7.49–7.48 (m, 1H), 6.93 (s, 1H), 4.48–4.44 (q, 2H, *J* = 7.2Hz), 2.31 (s, 3H), 1.44–1.41 (t, 3H, *J* = 7.2Hz); ^13^C-NMR (150 MHz CDCl_3_) *δ* = 186.71, 182.49, 164.57, 139.18, 138.16, 137.64, 134.20, 132.70, 132.03, 130.55, 128.78, 127.77, 123.81, 118.96, 111.33, 108.23, 93.33, 60.23, 29.66, 14.98, 14.45; LC–MS (ESI, Positive): m/z: (M79Br)^+^, and (M81Br)^+^ = 413, and 415, respectively; Anal. calculated for: C_20_H_16_NBrO_4_; C, 57.99; H, 3.89; N, 3.38; Found: C, 57.93; H, 3.87; N, 3.43.

#### Methyl 3-(4-Fluorobenzoyl)-7-methyl-2-phenylindolizine-1-carboxylate (5)

Appearance: Yellow crystalline compound; FT–IR (KBr, cm^–1^): 2972.10, 1685.67, 1602.74, 1379.01, 1217.00, 1118.64; ^1^H-NMR (400 MHz CDCl_3_) *δ* = 9.54–9.52 (d, 1H, *J* = 7.2 Hz), 8.25 (s, 1H), 7.37–7.34 (m, 2H), 7.10–7.02 (m, 5H), 6.92–6.91 (m, 1H), 6.70–6.66 (m, 2H), 3.70 (s, 3H), 2.53 (s, 3H); ^13^C-NMR (150 MHz CDCl_3_) *δ* = 186.54, 165.33, 164.88, 162.83, 140.71, 139.76, 139.03, 135.72, 135.69, 133.86, 131.52, 131.43, 131.16, 127.61, 127.25, 126.89, 121.87, 118.49, 117.48, 114.53, 114.31, 103.48, 50.81, 21.64; LC–MS (ESI, Positive): m/z: 388 (M + H)^+^; Anal. calculated for: C_24_H_18_NFO_3_; C, 74.41; H, 4.68; N, 3.62; Found: C, 74.43; H, 4.67; N, 3.63.

### 3.3. Anti-Tubercular Activity by Resazurin Microplate Assay (REMA)

The anti-TB properties of test compounds 1a–e, 2a–e, 3a–e, 4, and 5 were evaluated against the susceptible H37Rv and MDR strains of *Mycobacterium tuberculosis* using the colorimetric REMA plate method [38]. The detailed test sample preparation, anti-TB screening procedure, and the determination of the minimum inhibitory concentration (MIC) are described in a previous work [30]. The MICs of the test compounds were defined as the minimum drug concentrations needed to inhibit the growth of the organism with no color changes present in the 96-well microplate.

### 3.4. Safety Studies

Title compounds 2d, 2e, and 4, which exhibited the promising anti-TB activity against H37Rv and MDR strains of *Mycobacterium tuberculosis*, were subjected to safety studies by the 3-(4,5-dimethylthiazol-2-yl)-2,5-diphenyltetrazolium bromide (MTT) assay against peripheral blood mononuclear cells (PBMCs) according to the described protocol [39].

### 3.5. Computational Studies

The molecular modeling protocol, as reported, to our recent paper [14], was applied and carried out using Accelrys Discovery Studio 4.0, employing the CHARMm force fields algorithm. In the docking study, the X-ray co-crystal structures of the ligand and enzyme (Protein Data Bank (PDB) code: 4B6C, 5CBB, 5OP9) were used to evaluate the binding affinity of new indolizines. Prior to the docking studies, the protein was retrieved from the PDB and subjected to the following sequences: ligands and water exclusion, protein preparation, and minimization energy, and the concerned ligand insertion into the minimized protein to select the cavity site responsible for the binding domain. Molecular docking was conducted according to the CDocker protocol, insofar as the flexible ligand was docked into the rigid receptor. Docking scores of the ten best conformations were reported as CDocker energy and CDocker interaction energy and ranked according to CDocker energy. The stronger, negative CDocker scores indicated a more favorable binding interaction. Additional scoring functions (PLP1, PLP2, Jain, and PMF) were further examined to ensure the optimal ligand orientation into the receptor’s active binding site. The highest negative score of PLP1, PLP2, Jain, and PMF, indicating the strongest receptor–ligand-binding affinities, was considered to refine the binding pose.

## 4. Conclusions

The inhibitory activity of diversely substituted indolizines 1a–e, 2a–e, 3a–e, 4, and 5 against H_37_Rv and MDR strains of MTB were reported. Our medicinal chemistry program toward the discovery of novel anti-TB compounds enabled the identification of key substituents in the indolizine scaffold, which is responsible for generating a bioactive compound. The functionalization of the indolizine moiety at the C-7 and C-2 position by methyl/formyl groups and a methyl substituent, respectively—as well as the presence of halogen in the para position of the benzoyl ring at position C-3—were essential for displaying significant anti-TB activity. However, the presence of a phenyl group at the C-3 position led to lower anti-TB activity. Among the indolizine series, compound 4 was the most active, as it exhibited a MIC value of 4 µg/mL against susceptible H37Rv strains of MTB. Importantly, compounds 2d, 2e, and 4 were observed to exert anti-TB activity against MTB clinical isolates with multi-resistance to first-line anti-TB agents (rifampicin and isoniazid). A safety study performed by the MTT assay revealed that up to 500 µg/mL of active compounds 2d, 2e, and 4 were nontoxic across PBM cell lines. Computational docking analysis led to the identification of three potential molecular targets, namely CYP121, malate synthase, and GyrB ATPase. Efforts will be pursued in designing novel indolizines through a structure-based design approach with the aim of elucidating their mechanism of action. Based on our findings, and on the structural diversity of the compound, indolizine represents a promising scaffold for future development for anti-TB drug discovery.

## Figures and Tables

**Table 1:** Physicochemical characteristics of ethyl 3-(4-substitutedbenzoyl)-7-substituted-2-substitutedindolizine-1-carboxylates (4 and 5).

Entry	Mol Formula (Mol Mass)	R^1^	R^2^	R^3^	Yield (%) *^a^*	m.p. (°C)	cLog*P ^b^*
4	C_20_H_16_NO_4_Br (413)	CHO	CH_3_	Br	86	105–106	5.2033
5	C_24_H_18_NO_3_F (387)	CH_3_	C_6_H_5_	F	88	167–168	6.3602

*^a^* Yield of the product was calculated after column chromatography purification. *^b^* cLog*P* of the title compounds was calculated using ChemDraw Professional 16.0. m.p.: melting point.

**Table 2:** In vitro anti-TB activity of indolizines 1a–e, 2a–e, 3a–e, 4, and 5 against susceptible H37Rv and multi-drug-resistant (MDR) strains of *Mycobacterium tuberculosis* (MTB)

Entry	R^1^	R^2^	R^3^	MIC (µg/mL)	
				H37Rv	MDR–MTB ^b^
1a ^a^	CH_3_	H	H	>32	-
1b ^a^	CH_3_	H	F	>32	-
1c ^a^	CH_3_	H	Cl	>32	-
1d ^a^	CH_3_	H	Br	>32	-
1e ^a^	CH_3_	H	CN	>32	-
2a ^a^	CH_3_	CH_3_	H	>32	-
2b ^a^	CH_3_	CH_3_	F	>32	-
2c ^a^	CH_3_	CH_3_	Cl	16 ± 0.02 ^c,e^	>32
2d ^a^	CH_3_	CH_3_	Br	16 ± 0.02 ^c,e^	32 ± 0.02 ^c,e^
2e ^a^	CH_3_	CH_3_	CN	16 ± 0.02 ^d,e^	32 ± 0.02 ^c,d^
3a ^a^	CH_3_	CH_2_CH_3_	H	16 ± 0.02 ^c,d^	>32
3b ^a^	CH_3_	CH_2_CH_3_	F	>32	-
3c ^a^	CH_3_	CH_2_CH_3_	Cl	>32	-
3d ^a^	CH_3_	CH_2_CH_3_	Br	>32	-
3e ^a^	CH_3_	CH_2_CH_3_	CN	>32	-
4	CHO	CH_3_	Br	4 ± 0.02 ^c,e^	32 ± 0.02 ^c,e^
5	CH_3_	C_6_H_5_	F	32 ± 0.02 ^c,d^	>32

Note: MIC (minimum inhibitory concentration). ^a^ Synthesis and structural elucidation of the indolizine compounds are reported in [21]. ^b^ MDR-MTB isolates were found to be resistant to first-line antibiotics, isoniazid (0.2 µg/mL), and rifampicin (1 µg/mL). ^c–e^ The compounds not sharing a letter differ significantly (*p* < 0.05).

**Table 3:** Docking results of the indolizine derivatives 2c–e, 3a–e, 4, and 5 into the binding domain of the selected molecular targets.

Entry	CYP 121A1 (PDB 5OP9)			Malate Synthase (PDB 5CBB)			DNA GyrB ATPase (PDB 4B6C)		
	CDocker E. (Kcal/mol)	Residue Interactions		CDocker E. (Kcal/mol)	Residue Interactions		CDocker E. (Kcal/mol)	Residue Interactions	
		H-Bond (Dist. Å, atom)	Pi-Bond		H-Bond (Dist. Å, atom)	Pi-Bond		H-Bond (Dist. Å, atom)	Pi-Bond
2c	−42	Gln 385 (2.07, CO benzoyl)	Phe168	−44	Arg 339 (2.72, CO benzoyl) Phe 460 (1.90, CO ester) Leu 461 (2.57, CO ester)	Try 541	−34	Gln 102 (2.27, CO benzoyl)	Glu 56
2d	−42	Gln 385 (1.90, CO benzoyl)	Phe168	−45	Arg 339 (2.71, CO benzoyl) Phe 460 (1.99, CO ester)	Try 541	−33	Gln 102 (2.32, CO benzoyl)	Glu 56
2e	−41	Gln 385 (1.98, CO benzoyl)	Phe168	−41	Phe 460 (2.48, CO ester) Asp 633 (1.83, CO benzoyl)	Try 541	−37	Gln 102 (2.16, CO ester)	Arg 82
3a	−38	Gln 385 (2.27, CO benzoyl)	Phe168	−31	Arg 339 (2.36, CO benzoyl)	Asp 462	−34	Gln 102 (2.21, CO benzoyl)	Arg 82
3b	−41	His 343 (2.28, F)	Phe168	−36	Arg 339 (2.62, CO benzoyl) Phe 460 (2.31, CO ester) Leu 461 (2.68, CO ester) Asp 462 (2.93, CO ester)	Try 541	−33	Gln 102 (2.45, CO benzoyl)	Arg 82
3c	−41	Gln 385 (2.21, CO benzoyl)	Met 62	−40	Arg 339 (2.74, CO benzoyl) Phe 460 (1.94, CO ester) Leu 461 (2.62, CO ester)	Try 541	−34	Gln 102 (2.45, CO benzoyl)	Glu 56
3d	−41	Gln 385 (2.24, CO benzoyl)	Met 62	−37	Arg 339 (2.56, CO benzoyl) Phe 460 (2.25, CO ester)) Leu 461 (2.64, CO ester) Asp 462 (2.94, CO ester)	Try 541	−33	Gln 102 (2.45, CO benzoyl)	Arg 82
3e	−39	Gln 385 (2.94, CN)	-	−36	Arg 339 (2.55, CO benzoyl) Phe 460 (2.14, CO ester) Leu 461 (2.44, CO ester) Asp 462 (2.92, CO ester)	Try 541	−33	Gln 102 (2.26, CO benzoyl)	Arg 82
4	−44	Gln 385 Asn 85 (2.55, CO formyl)	Phe168	−50	Arg 339 (2.72, CO benzoyl) Phe 460 (1.91, CO ester) Leu 461 (2.57, CO ester) Arg 463 (2.34, CO formyl)	Try 541	−45	Gly 83 (2.13, CO formyl)Gln 102 (2.70, CO benzoyl) Arg 141 (3.04, CO ester)	Glu 56
5	−40	Gln 385 (2.02, CO benzoyl)	Phe168	−18	Arg 339 (2.32, CO benzoyl) Arg 463 (2.66, F) Asp 633 (2.37, O ester)	Asp 462	−27	-	Arg 82

## Supplementary Materials

The following are available online at https://www.mdpi.com/2079-6382/8/4/247/s1, Figure S1: FT–IR of ethyl 3-(4-bromobenzoyl)-7-formyl-2-methylindolizine-1-carboxylate (4); Figure S2: ^1^H-NMR of ethyl 3-(4-bromobenzoyl)-7-formyl-2-methylindolizine-1-carboxylate (4); Figure S3: ^13^C-NMR of ethyl 3-(4-bromobenzoyl)-7-formyl-2-methylindolizine-1-carboxylate (4); Figure S4: FT–IR of methyl 3-(4-fluorobenzoyl)-7-methyl-2-phenylindolizine-1-carboxylate (5); Figure S5: ^1^H-NMR of methyl 3-(4-fluorobenzoyl)-7-methyl-2-phenylindolizine-1-carboxylate (5); Figure S6: ^13^C-NMR of methyl 3-(4-fluorobenzoyl)-7-methyl-2-phenylindolizine-1-carboxylate (5).
