# Supplementary material for: Anti-Tubercular Activity of Substituted 7-Methyl and 7-Formylindolizines and In Silico Study for Prospective Molecular Target Identification
Source: Antibiotics (Basel). 2019 Dec 3;8(4):247. doi: 10.3390/antibiotics8040247 (PMC6963442; doi:10.3390/antibiotics8040247)
Supplement: Supplementary file 1 [file antibiotics-08-00247-s001.pdf]

antibiotics

Anti-Tubercular Activity of Substituted 7-Methyl and 7-Formylindolizines and *in Silico* Study for Prospective Molecular Target Identification

Katharigatta N. Venugopala [1,2,*], Christophe Tratrat [1], Melendhran Pillay [3], Fawzi M. Mahomoodally [4], Subhrajyoti Bhandary [5], Deepak Chopra [5], Mohamed A. Morsy [1,6], Michelyne Haroun [1], Bandar E. Aldhubiab [1], Mahesh Attimarad [1], Anroop B. Nair [1], Nagaraja Sreeharsha [1], Rashmi Venugopala [7], Sandeep Chandrashekharappa [8,*], Osama I. Alwassil [9], Bharti Odhav [2]

1 Department of Pharmaceutical Sciences, College of Clinical Pharmacy, King Faisal University, Al-Ahsa 31982, Kingdom of Saudi Arabia; kvenugopala@kfu.edu.sa (K.N.V.); ctratrat@kfu.edu.sa (C.T.); momorsy@kfu.edu.sa (M.A.M.) mharoun@kfu.edu.sa (M.H.); baldhubiab@kfu.edu.sa (B.E.A.); mattimarad@kfu.edu.sa (M.A.); anair@kfu.edu.sa (A.B.N.); sharsha@kfu.edu.sa (N.S.)

2 Department of Biotechnology and Food Technology, Durban University of Technology, Durban 4001, South Africa; odhavb@dut.ac.za (B.O.)

3 Department of Microbiology, National Health Laboratory Services, KZN Academic Complex, Inkosi Albert Luthuli Central Hospital, Durban 4001, South Africa; melendhra.pillay@nhls.ac.za

4 Department of Health Sciences, Faculty of Science, University of Mauritius, Réduit, Mauritius; f.mahomoodally@uom.ac.mu (F.M.M.)

5 Department of Chemistry, Indian Institute of Science Education and Research Bhopal, Indore By-pass Road, Bhauri, Bhopal 462 066, Madhya Pradesh, India; subhb@iiserb.ac.in (S.B); dchopra@iiserb.ac.in (D.C.)

6 Department of Pharmacology, Faculty of Medicine, Minia University, El-Minia 61511, Egypt

7 Department of Public Health Medicine, University of KwaZulu-Natal, Howard College Campus, Durban 4001, South Africa; venugopalar@ukzn.ac.za (R.V.)

8 Institute for Stem Cell Biology and Regenerative Medicine, NCBS, TIFR, GKVK, Bellary Road, Bangalore 560 065, India; sandeepc@instem.res.in (S.C.)

9 Department of Pharmaceutical Sciences, College of Pharmacy, King Saud bin Abdulaziz University for Health Sciences, Riyadh, Kingdom of Saudi Arabia; wassilo@ksau-hs.edu.sa (O.I.A.)

* Correspondence: kvenugopala@kfu.edu.sa; Tel.: +966 1358 98842 (K.N.V.); sandeepc@instem.res.in; Tel.: +91 94 4863 9413 (S.C)

Table of Contents.

Sl. No.	Contents	Page No.
**S1**	FT-IR of ethyl 3-(4-bromobenzoyl)-7-formyl-2-methylindolizine-1-carboxylate (**4**)	**3**
**S2**	^{1}H-NMR of ethyl 3-(4-bromobenzoyl)-7-formyl-2-methylindolizine-1-carboxylate (**4**)	**4**
**S3**	^{13}C-NMR of ethyl 3-(4-bromobenzoyl)-7-formyl-2-methylindolizine-1-carboxylate (**4**)	**5**
**S4**	FT-IR of methyl 3-(4-fluorobenzoyl)-7-methyl-2-phenylindolizine-1-carboxylate (**5**)	**6**
**S5**	^{1}H-NMR of methyl 3-(4-fluorobenzoyl)-7-methyl-2-phenylindolizine-1-carboxylate (**5**)	**7**
**S6**	^{13}C-NMR of methyl 3-(4-fluorobenzoyl)-7-methyl-2-phenylindolizine-1-carboxylate (**5**)	**8**

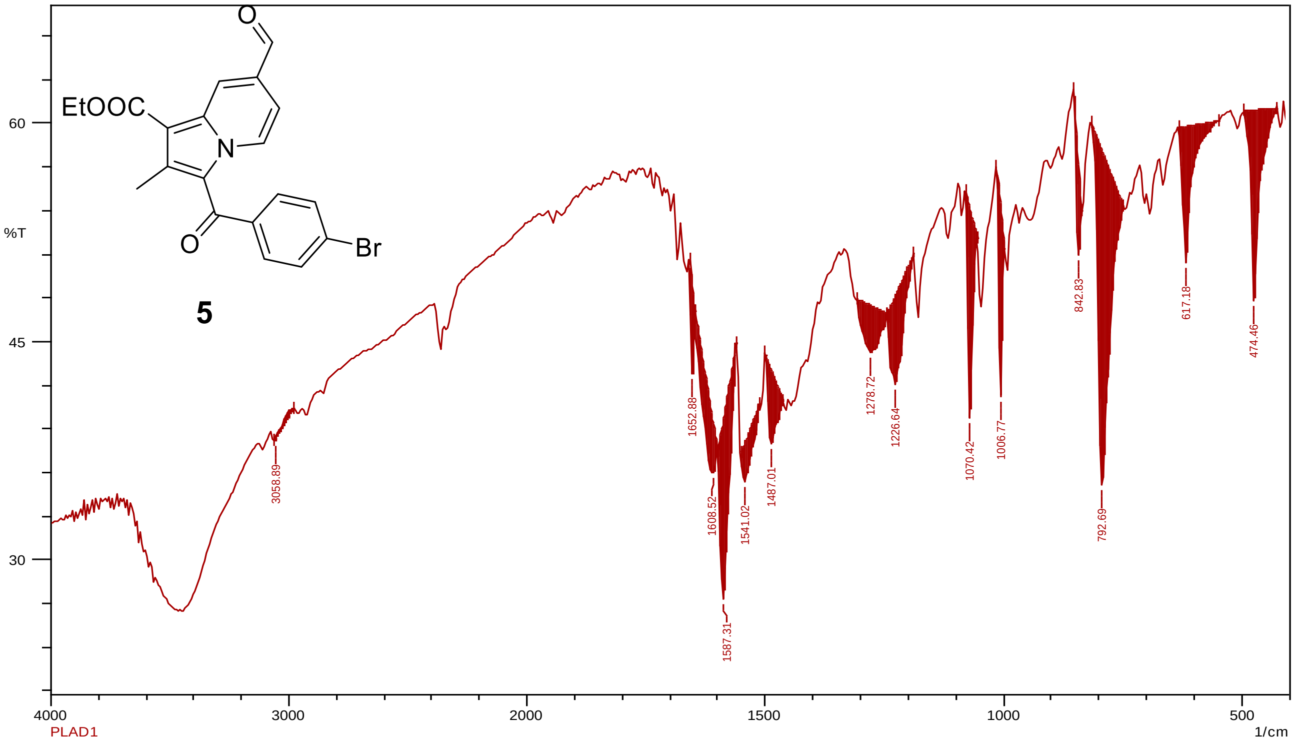

S1: FT-IR of ethyl 3-(4-bromobenzoyl)-7-formyl-2-methylindolizine-1-carboxylate (**4**).

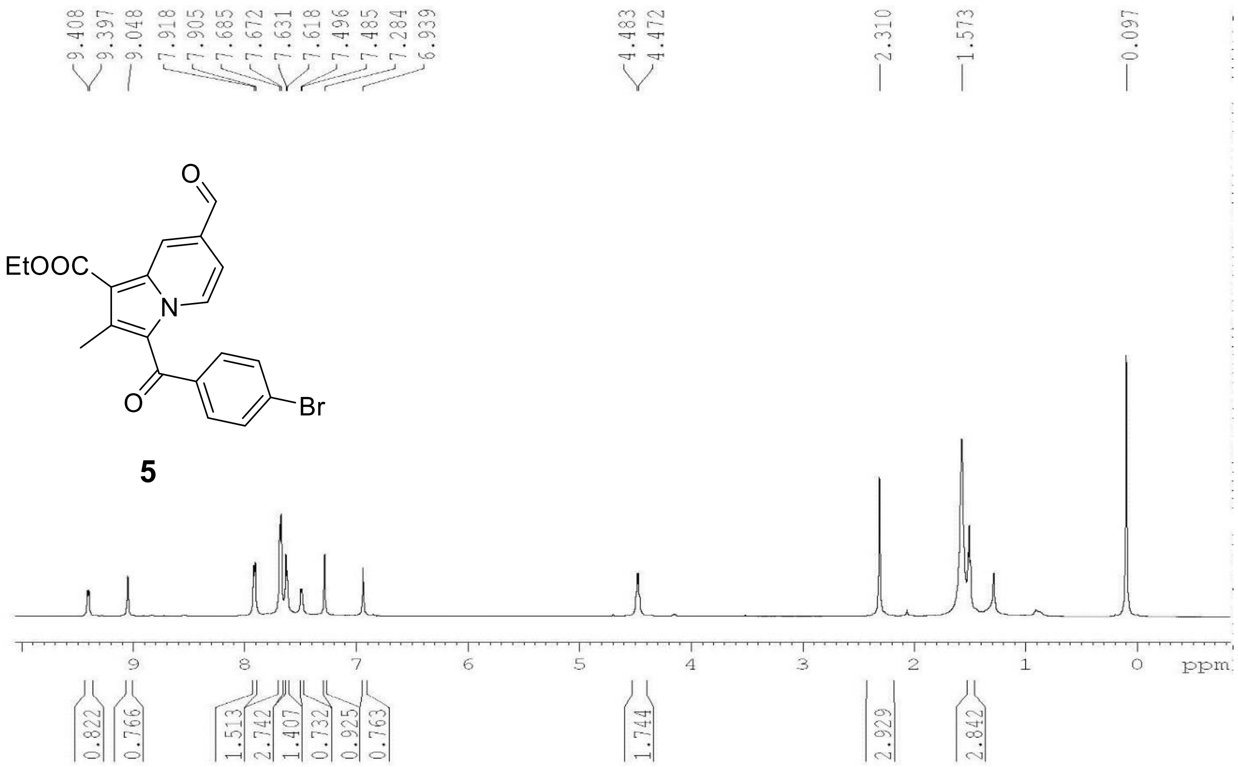

S2: ^{1}H-NMR of ethyl 3-(4-bromobenzoyl)-7-formyl-2-methylindolizine-1-carboxylate (**4**).

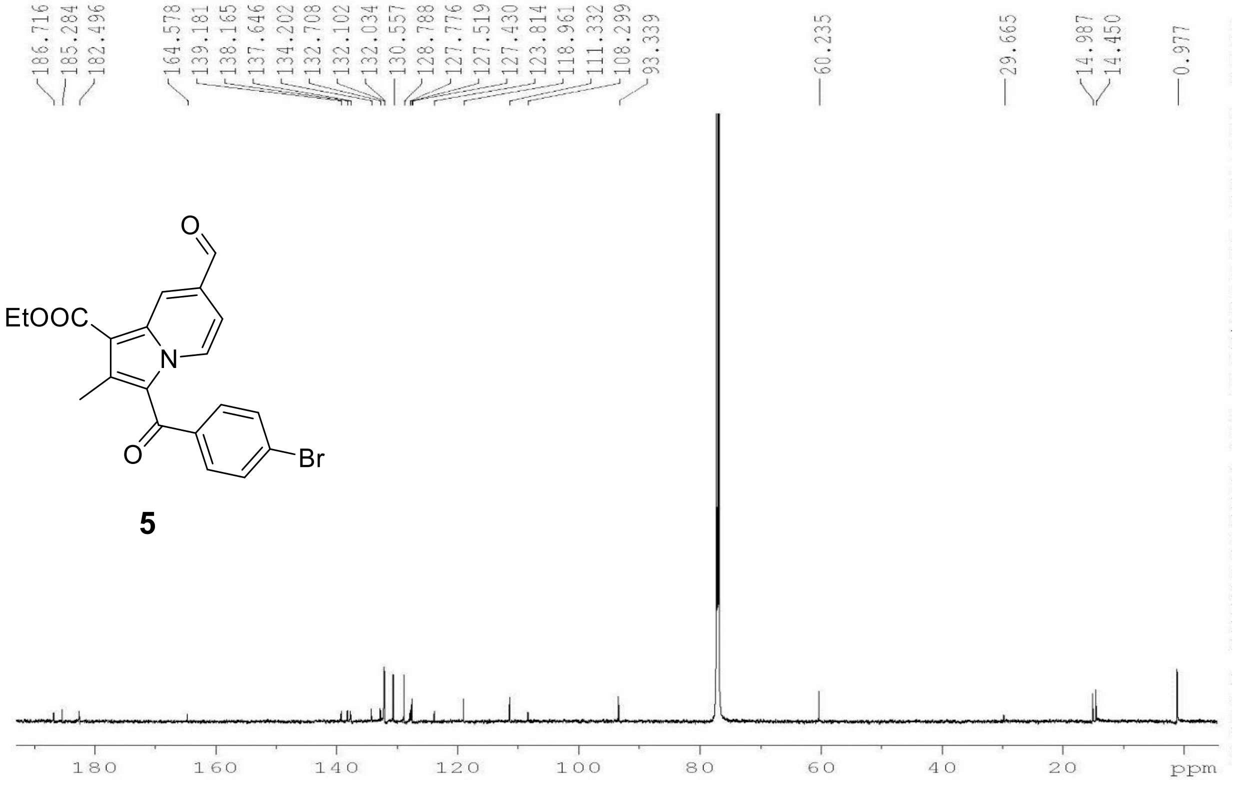

S3: ^{13}C-NMR of ethyl 3-(4-bromobenzoyl)-7-formyl-2-methylindolizine-1-carboxylate (**4**).

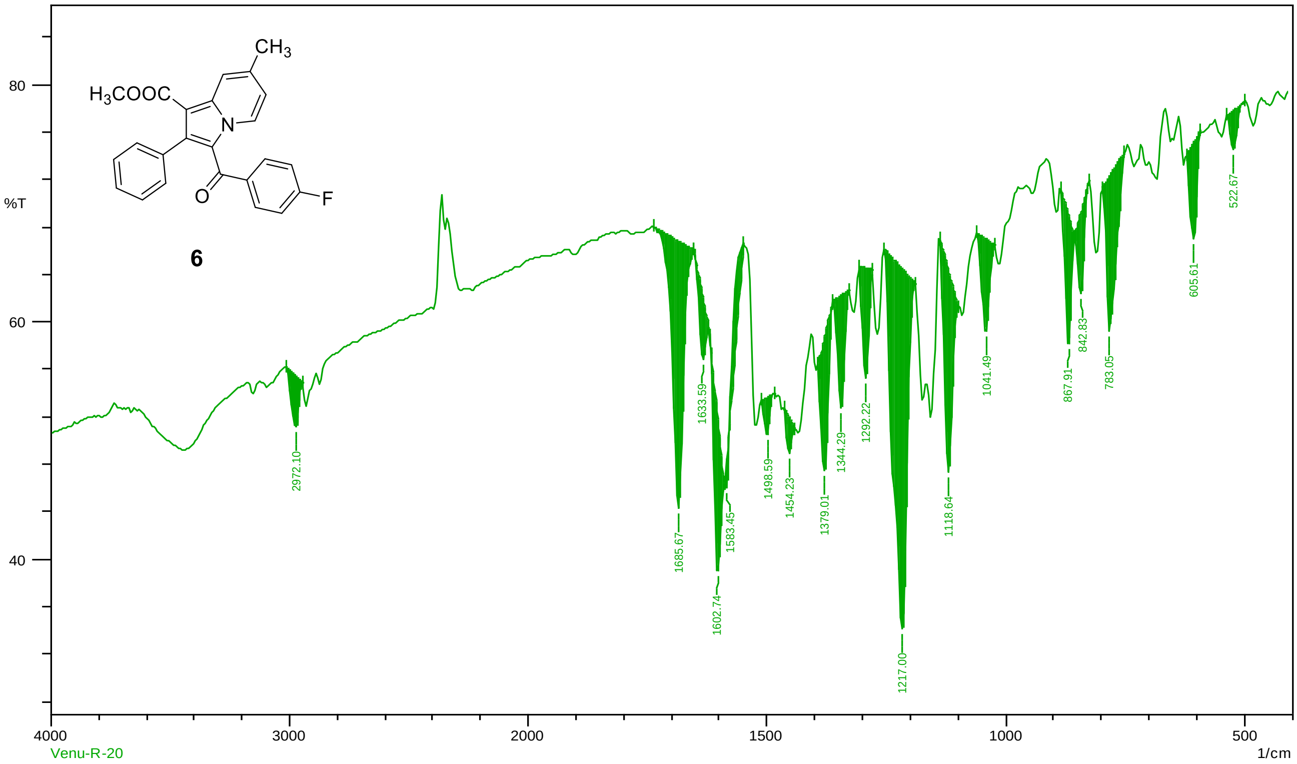

S4: FT-IR of methyl 3-(4-fluorobenzoyl)-7-methyl-2-phenylindolizine-1-carboxylate (**5**).

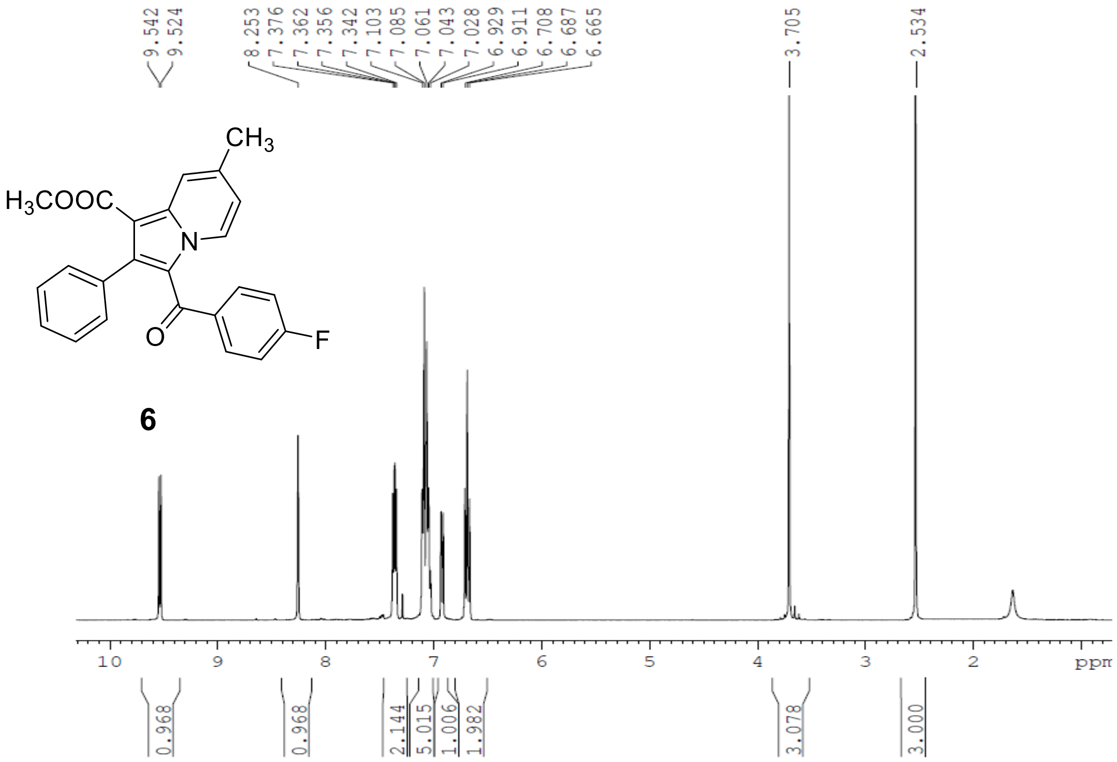

S5: ^{1}H-NMR of methyl 3-(4-fluorobenzoyl)-7-methyl-2-phenylindolizine-1-carboxylate (**5**).

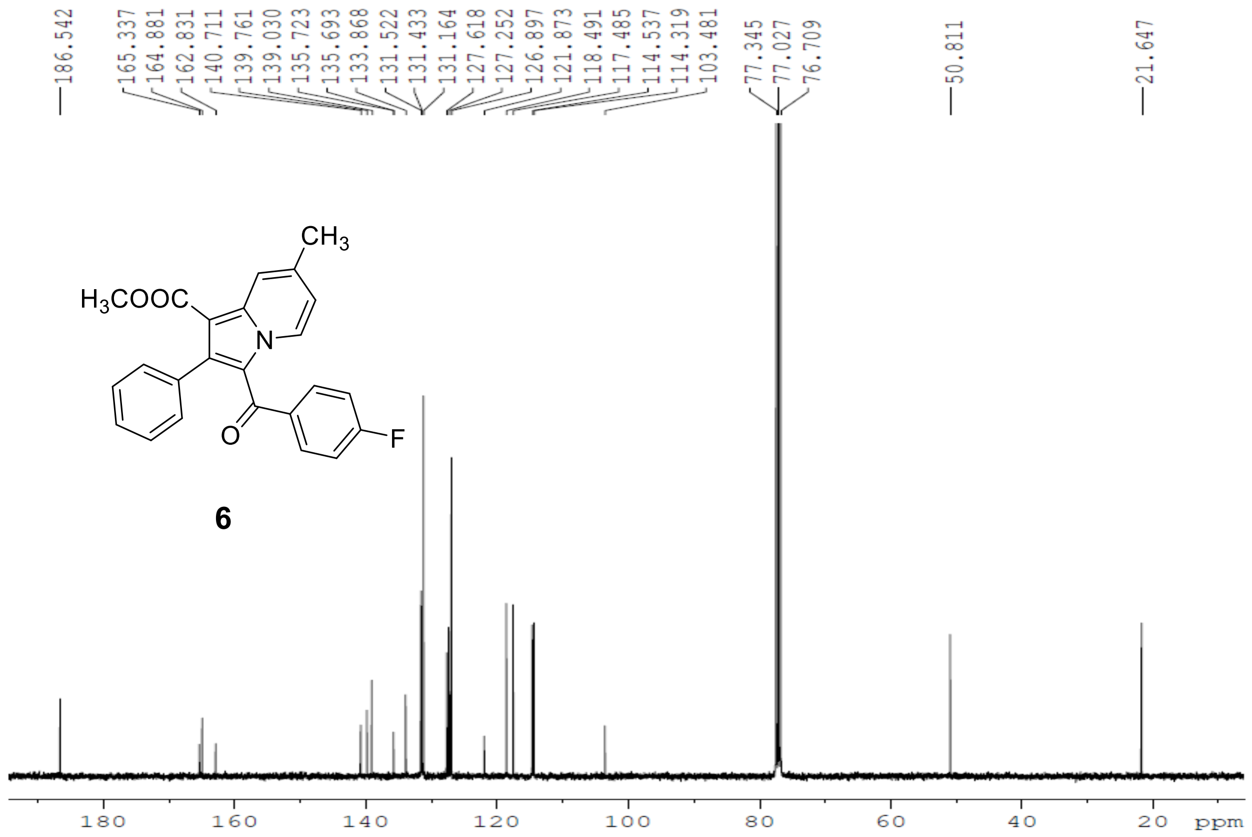

S6: ^{13}C-NMR of methyl 3-(4-fluorobenzoyl)-7-methyl-2-phenylindolizine-1-carboxylate (5).
